# Rapid *Leptospira *identification by direct sequencing of the diagnostic PCR products in New Caledonia

**DOI:** 10.1186/1471-2180-10-325

**Published:** 2010-12-22

**Authors:** Julie Perez, Cyrille Goarant

**Affiliations:** 1 Institut Pasteur de Nouvelle-Calédonie, Réseau International des Instituts Pasteur, Laboratoire de Recherche en Bactériologie, BP61, 98845 Nouméa cedex, New Caledonia

## Abstract

**Background:**

Most of the current knowledge of leptospirosis epidemiology originates from serological results obtained with the reference Microscopic Agglutination Test (MAT). However, inconsistencies and weaknesses of this diagnostic technique are evident. A growing use of PCR has improved the early diagnosis of leptospirosis but a drawback is that it cannot provide information on the infecting *Leptospira *strain which provides important epidemiologic data. Our work is aimed at evaluating if the sequence polymorphism of diagnostic PCR products could be used to identify the infecting *Leptospira *strains in the New Caledonian environment.

**Results:**

Both the *lfb1 *and *secY *diagnostic PCR products displayed a sequence polymorphism that could prove useful in presumptively identifying the infecting leptospire. Using both this polymorphism and MLST results with New Caledonian isolates and clinical samples, we confirmed the epidemiological relevance of the sequence-based identification of *Leptospira *strains. Additionally, we identified one cluster of *L. interrogans *that contained no reference strain and one cluster of *L. borgpetersenii *found only in the introduced Rusa deer *Cervus timorensis russa *that is its probable reservoir.

**Conclusions:**

The sequence polymorphism of diagnostic PCR products proved useful in presumptively identifying the infecting *Leptospira *strains. This could contribute to a better understanding of leptospirosis epidemiology by providing epidemiological information that cannot be directly attained from the use of PCR as an early diagnostic test for leptospirosis.

## Background

Leptospirosis is recognized as the most widespread zoonosis worldwide [1]. It can be a lethal disease with high endemicity in the tropics. However, epidemics have also been described, most frequently associated with particular meteorological events [2,3].

The epidemiology of leptospirosis has classically been described on the basis of serological data, an indirect biomarker, using the Microscopic Agglutination Test (MAT), a technique regarded so far as the "gold standard" for identifying the infecting serovar from human or animal sera [1,4]. MAT results have provided epidemiologically important data allowing the identification of the infection sources or reservoirs and have largely contributed to the current knowledge of leptospirosis epidemiology. However, MAT is not without weaknesses and was notably shown to be a poor predictor of the infection serovar [5].

The taxonomy of the genus *Leptospira *has now been clarified from genetics and leptospirosis can now be studied using genetic tools, when isolates are available [6,7]. Similarly, leptospirosis diagnosis increasingly relies on PCR results [3], where a single positive sample provides a certainty diagnosis before serological conversion [4]. This frequently results in the loss of the serology-based identification of the infecting strains, which is epidemiologically important to identify the reservoirs. Therefore, the increased use of PCR has greatly improved the early diagnosis of leptospirosis, but paradoxically restricts data available for epidemiological surveillance. Yet, because the genetic tools implemented provide an insight into the genome of the infecting strain, epidemiologically relevant information might be deduced from sequence polymorphisms of the diagnostic PCR products. This approach was notably suggested and evaluated by Victoria et al. [8] while studying the phylogeny of the *S10-spc-α *locus: these authors demonstrated that this locus is highly conserved and a useful phylogenic target. They additionally suggested a short 245 bp region of *secY *as a suitable target for diagnosing leptospirosis by PCR, the sequence of the diagnostic PCR product then being epidemiologically informative. Actually, a diagnostic PCR using this target was later designed, validated according to international guidelines and confirmed to provide an epidemiologically relevant phylogeny [9].

New Caledonia is an archipelago of the South-West Pacific (19-23°S; 164-167°E). Leptospirosis is known to be endemic with epidemic bursts occurring during hot rainy periods [3,10-12]. Presumptive serovars in New Caledonia based on MAT on human leptospirosis cases are Copenhageni, Icterohaemorragiae, Castellonis, Panama, Pomona, Australis and Pyrogenes [10,11,13,14]. The only native mammals are bats and flying foxes. Very few imported mammals are present: 4 rodent species (*Rattus rattus*, *Rattus norvegicus*, *Rattus exulans *and *Mus musculus*) and domestic as well as feral dogs, cats, cattle, horses, goats, sheeps and the Rusa deer *Cervus timorensis russa*.

The qPCR technique used for leptospirosis diagnosis in New Caledonia amplifies a 331pb DNA fragment within the *lfb1 *gene, which sequence polymorphism allows the identification of the species of the infecting *Leptospira *strain using melting curve analysis [15].

The Multi Locus Sequence Typing (MLST) technique uses sequence polymorphisms of multiple housekeeping genes for isolate characterization and to investigate evolutionary relationships among closely-related bacteria. It is increasingly considered as the gold standard typing method, at least in species where sufficient sequence polymorphisms exists in housekeeping genes, because it relies on sequence data that are exchangeable and independent of the analytical platform [16,17]. This technique, successfully applied to a number of bacterial pathogens, was notably recently applied to the study of leptospires: various typing schemes based on the comparison of 2855-3165 bp concatenated sequences of housekeeping genes were proposed [18-20] and evaluated over *Leptospira *spp. reference strains and isolates.

Because of the limited mammal diversity in New Caledonia, we hypothesized that a limited diversity of pathogenic *Leptospira *strains would be present and aimed at evaluating if the sequence polymorphism of diagnostic PCR products would allow the identification of the infecting *Leptospira*. To better investigate this hypothesis and the epidemiology of leptospirosis in New Caledonia, we also performed a MLST study on a collection of isolates and evaluated its direct feasibility using leptospirosis patients' serum DNA extracts. Additionally, extracts from *Leptospira*-infected deer kidneys contributed to a better description of the *Leptospira *strains currently involved in leptospirosis in New Caledonia.

## Methods

### Bacterial strains

The strains studied were collected from 1989 to 2000 throughout mainland New-Caledonia. Eighteen were isolates from patients' blood received at Institut Pasteur for diagnosis purpose, and 2 were isolated from deer in 1992, kindly provided by the New Caledonian Reference Veterinary Laboratory. Previously studied VNTR (Variable Nucleotide Tandem Repeat) profiles and serological identification of these isolates [13] allowed the selection of isolates from the 4 different serovars identified in our collection. The list of the isolates, their serological and VNTR-based identifications are presented in Table 1.

**Table 1:** New Caledonian Leptospira isolates analyzed in the present study.

Isolate	Species	Serogroup	**VNTR-based serovar **[13]	Source
1989-01	*L. interrogans*	Icterohaemorragiae	Copenhageni or Icterohaemorragiae	human

1995-06	*L. interrogans*	Icterohaemorragiae	Copenhageni or Icterohaemorragiae	human

1989-07	*L. interrogans*	Icterohaemorragiae	Copenhageni or Icterohaemorragiae	human

1995-09	*L. interrogans*	Icterohaemorragiae	Copenhageni or Icterohaemorragiae	human

2000-14	*L. interrogans*	Icterohaemorragiae	Copenhageni or Icterohaemorragiae	human

1995-01	*L. interrogans*	Pomona	Pomona	human

1989-03	*L. interrogans*	Pomona	Pomona	human

1997-05	*L. interrogans*	Pomona	Pomona	human

1990-17	*L. interrogans*	Pomona	Pomona	human

LTDV15	*L. interrogans*	Pomona	Pomona	deer (1992)

1993-01	*L. interrogans*	Pyrogenes	unidentified	human

1993-04	*L. interrogans*	Pyrogenes	unidentified	human

1995-04	*L. interrogans*	Pyrogenes	unidentified	human

1999-07	*L. interrogans*	Pyrogenes	unidentified	human

1989-08	*L. interrogans*	Pyrogenes	unidentified	human

1995-03	*L. borgpetersenii*	Ballum	Castellonis	human

1999-12	*L. borgpetersenii*	Ballum	Castellonis	human

1990-13	*L. borgpetersenii*	Ballum	Castellonis	human

1990-14	*L. borgpetersenii*	Ballum	Castellonis	human

LTDV14	*L. borgpetersenii*	Sejroe	Hardjo (type Hardjo-bovis)	deer (1992)

GenBank accession numbers of the sequences obtained from these isolates are provided as additional file 1 Table S1.

### Clinical specimens

Clinical samples (sera) routinely received at Institut Pasteur in Nouméa, for the diagnosis of leptospirosis were also included in the study. We studied 88 human PCR positive sera collected from January 2008 to February 2010. Twelve PCR-positive deer kidney samples collected in 2010 during a sampling campaign in a slaughterhouse were also included. The 27 human samples used for drawing phylogenic trees are summarized in Table 2.

**Table 2:** Clinical specimens analyzed in the present study.

Specimen identification	Source	***Leptospira *concentration based on qPCR **[15]	*lfb1*-based cluster (see results)
08323250	Human serum	< 50/ml	*L. borgpetersenii *1

08238362	Human serum	< 50/ml	*L. interrogans *3

09022251	Human serum	< 50/ml	*L. interrogans *2

09037333	Human serum	< 50/ml	*L. interrogans *3

09046172	Human serum	< 50/ml	*L. interrogans *2

09068284	Human serum	< 50/ml	*L. borgpetersenii *1

09106497	Human serum	< 50/ml	*L. interrogans *2

09110512	Human serum	< 50/ml	*L. interrogans *4

09139265	Human serum	< 50/ml	*L. borgpetersenii *1

09162317	Human serum	< 50/ml	*L. borgpetersenii *1

09337238	Human serum	< 50/ml	*L. interrogans *3

10032221	Human serum	< 50/ml	*L. borgpetersenii *1

10073167	Human serum	< 50/ml	*L. interrogans *1

08099430	Human serum (fatal case)	50/ml	*L. interrogans *2

09073008	Human serum	50/ml	*L. interrogans *2

09131462	Human serum	55/ml	*L. borgpetersenii *1

09117472	Human serum	60/ml	*L. borgpetersenii *1

09233024	Human serum	200/ml	*L. interrogans *1

08121411	Human serum	320/ml	*L. interrogans *4

09100462	Human serum	320/ml	*L. interrogans *5

09031188	Human serum	920/ml	*L. interrogans *1

08095345	Human serum (fatal case)	1100/ml	*L. interrogans *1

09043326	Human serum	1100/ml	*L. interrogans *5

09210289	Human serum	1100/ml	*L. interrogans *5

09145359	Human serum	1600/ml	*L. interrogans *1

09044463	Human serum (fatal case)	5800/ml	*L. interrogans *5

09243410	Human serum (fatal case)	6300/ml	*L. interrogans *1

Deer 16	Deer kidney	< 50/mg	*L. borgpetersenii *2

Deer 39	Deer kidney	< 50/mg	*L. interrogans *1

Deer 3	Deer kidney	50/mg	*L. interrogans *4

Deer 10	Deer kidney	80/mg	*L. borgpetersenii *2

Deer 13	Deer kidney	82/mg	*L. interrogans *1

Deer 9	Deer kidney	88/mg	*L. borgpetersenii *2

Deer 14	Deer kidney	300/mg	*L. borgpetersenii *2

Deer 15	Deer kidney	675/mg	*L. borgpetersenii *2

Deer 21	Deer kidney	625/mg	*L. borgpetersenii *2

Deer 2	Deer kidney	1100/mg	*L. interrogans *4

Deer 27	Deer kidney	3700/mg	*L. interrogans *4

GenBank accession numbers of the sequences obtained from these specimens are provided in additional file 1 Table S2.

### DNA extraction

For human samples, total DNA from serum (200 μl) was extracted using an automatic method on an EasyMAG apparatus (Biomerieux). For bacterial cultures and animal samples, total DNA from a culture pellet, or kidney (ca. 25 mg) was extracted using the QIAamp DNA minikit (Qiagen) following the manufacturer's instructions.

### PCR analysis

The real time PCR routinely used for leptospirosis diagnosis targets the *lfb1 *gene as described by Mérien et al. [15] and was run on a LightCycler LC 2.0 using the LightCycler FastStart DNA Master SYBR Green I kit (Roche Applied Science, New Zealand).

For the MLST study, we used the typing scheme described by Thaipadungpanit *et al*. that uses the sequence polymorphism of *pntA*, *sucA*, *pfkB*, *tpiA*, *mreA*, *glmU *and *fadD *[20]. Amplifications were performed in a 25 μl total volume containing 1-10 ng genomic DNA, 5 pmol of each primer, 200 μM dNTP with 1.25 mM MgCl2. Two different DNA polymerases were used for DNA amplification: either 1 unit of Red Hot *Taq *DNA Polymerase, Thermo Scientific (ABgene) or 1.25 units of FastStart High Fidelity PCR System (Roche Applied Science), in their corresponding 1× buffer. A GeneAmp PCR system 9700 (Applied Biosystem) was used to perform PCR with an initial denaturation step at 94°C for 2 minutes, followed by 35 cycles of 94°C for 20 seconds, variable annealing temperature for 30 seconds, 72°C for 50 seconds for Red Hot *Taq *DNA Polymerase and 40 cycles of 94°C for 30 seconds, variable annealing temperatures for 30 seconds, 72°C for 50 seconds for FastStart High Fidelity DNA Polymerase, then 72°C for 7 minutes. PCR product size, primer sequences and annealing temperatures are shown in Table 3.

**Table 3:** Primers used in the present study

Name	Sequence	References	Target gene (amplicon size)	Annealing temperature (°C)
lfb1-F	CATTCATGTTTCGAATCATTTCAAA	[15]	*lfb1 *(331 bp)	60
			
lfb1-R	GGCCCAAGTTCCTTCTAAAAG			

secY-F	ATGCCGATCATTTTTGCTTC	[18]	*secY *(549 bp)	60
			
secY-R	AGTTGAGCCCGCAGTTTTC			

SecYIVF	GCGATTCAGTTTAATCCTGC	[9]	*secY *(202 bp)	54
			
SecYIV	GAGTTAGAGCTCAAATCTAAG			

pfkB-F	CCGAAGATAAGGGGCATACC	[20]	*pfkB *(559 bp)	52
			
pfkB-R	CAAGCTAAAACCGTGAGTGATT			
		
pntA-F	TGCCGATCCTACAACATTA		*pntA *(637 bp)	52
			
pntA-R	AAGAAGCAAGATCCACAACTAC			
		
sucA-F	AGAAGAGGCCGGTTATCATCAG		*sucA *(559 bp)	52
			
sucA-R	CTTCCGGGTCGTCTCCATTTA			
		
tpiA-F	AAGCCGTTTTCCTAGCACATTC		*tpiA *(554 bp)	52
			
tpiA-R	AGGCGCCTACAAAAAGACCAGA			
		
mreA-F	GTAAAAGCGGCCAACCTAACAC		*mreA *(601 bp)	45
			
mreA-R	ACGATCCCAGACGCAAGTAA			
		
glmU-F	GGAAGGGCACCCGTATGAA		*glmU *(556 bp)	50
			
glmU-R	TCCCTGAGCGTTTTGATTT			
		
fadD-F	AGTATGCGTATCTTCCTCCTT		*fadD *(576 bp)	50
			
fadD-R	TTCCCACTGTAATTTCTCCTAA			

The *secY *gene was also amplified using PCR conditions previously described [9,18] or combinations of forward and reverse primers of these 2 techniques. The recently described diagnostic PCR used the cycling conditions described by the authors [9], except that it was performed with the LightCycler FastStart DNA Master SYBR Green I kit on a LightCycler 2.0 apparatus and that the number of amplification cycles was increased to 50.

### Detection of PCR products

The amplification products were directly analyzed using the LightCycler software and/or visualized by gel electrophoresis in a 1.2% agarose gel stained with GelRed Nucleic Acid Gel Stain 1× (Biotium).

### DNA Sequencing

PCR products were purified using the MinElute PCR Purification Kit or MinElute Gel Extraction Kit (Qiagen) according to the manufacturer's instructions. Purified PCR products were directly sequenced in both forward and reverse directions using the same primers as for PCR using the ABI BigDye Terminator v3.1 cycle sequencing kit (Applied Biosystems) with the following modifications: Each 20 μl reaction contained 0.0625× ready reaction premix, 1× BigDye sequencing buffer, 3.2 pmol forward or reverse primer, 5-10 ng DNA and ddH_2_O. Cycle sequencing was performed using initial denaturation at 96°C for 1 minute followed by 60 cycles of 10 seconds at 96°C, 5 seconds at 50°C and 4 minutes at 60°C in a GeneAmp PCR System 9700 (Applied Biosystems). The sequencing products were purified on home-made Sephadex G-50 (Pharmacia) columns in Multiscreen filter plates (Millipore) and sequenced on an ABI 3730 × l automated sequencer.

Assembly, editing and finishing of the sequences using both the forward and reverse reaction results were made using the Staden Package [21]. DNA sequences from reference strains of relevant serovars were retrieved from http://www.mlst.net[20], from LepBank [22] or from GenBank.

### MLST data analysis

Individual gene or concatenate sequences were aligned using BioEdit version 7.0.9.0 [23]. Phylogenic analyses were conducted with PHYLO_WIN version 2 [24], the consensus tree being drawn based on 1000 bootstrap replicates with Kimura 2 parameter. *L. kirschneri *serovar Grippotyphosa was used as outgroup for all phylogenic analyses.

## Results

### PCR results on clinical isolates

All 7 PCRs described for the MLST scheme by Thaipadungpanit et al. [20] successfully amplified a product of the expected size with DNA from all isolates belonging to the species *L. interrogans*. However, for some isolates, the annealing temperature for amplifying *mreA *had to be lowered down to 45°C to obtain a successful amplification. For *L. borgpetersenii *isolates, only *pntA *and *glmU *could successfully be amplified. The *secY *product used by Ahmed et al. [18] was successfully amplified from all isolates, either *L. interrogans *or *L. borgpetersenii*. Using the diagnostic PCRs, *lfb1 *was amplified with extracts from human sera or deer kidney with leptospires concentration equal to or lower than 50 per ml or per mg, respectively. The *secY *diagnostic PCR product could be amplified from clinical samples containing down to ca. 60 leptospires/ml on our qPCR platform. *glmU *and *pntA *were successfully amplified from clinical specimens containing ≥ ca. 200 leptospires per ml using either DNA polymerase tested.

### Diagnostic PCR product-deduced phylogeny

We aimed at evaluating if the direct sequencing of a diagnostic PCR product could also allow the putative identification of the infecting strain. Early diagnosis of human leptospirosis in New Caledonia relies on the *lfb1 *PCR [15]. Therefore, the *lfb1 *diagnostic PCR products of the collection isolates, from patients recruited between January 2008 and February 2010 and from deer kidneys sampled in 2010 were directly sequenced. *lfb1 *sequences of reference strains retrieved from GenBank were also included and aligned. This allowed the construction of an *lfb1*-based phylogeny, supported by a 222 bp sequence. This allowed the distinction of 2 clusters among New Caledonian *L. borgpetersenii*-infected clinical samples, one including references sequences of the serovars Sejroe and Castellonis, the other including the sequence of the reference strain of Hardjo-bovis respectively.

**Figure 1:**
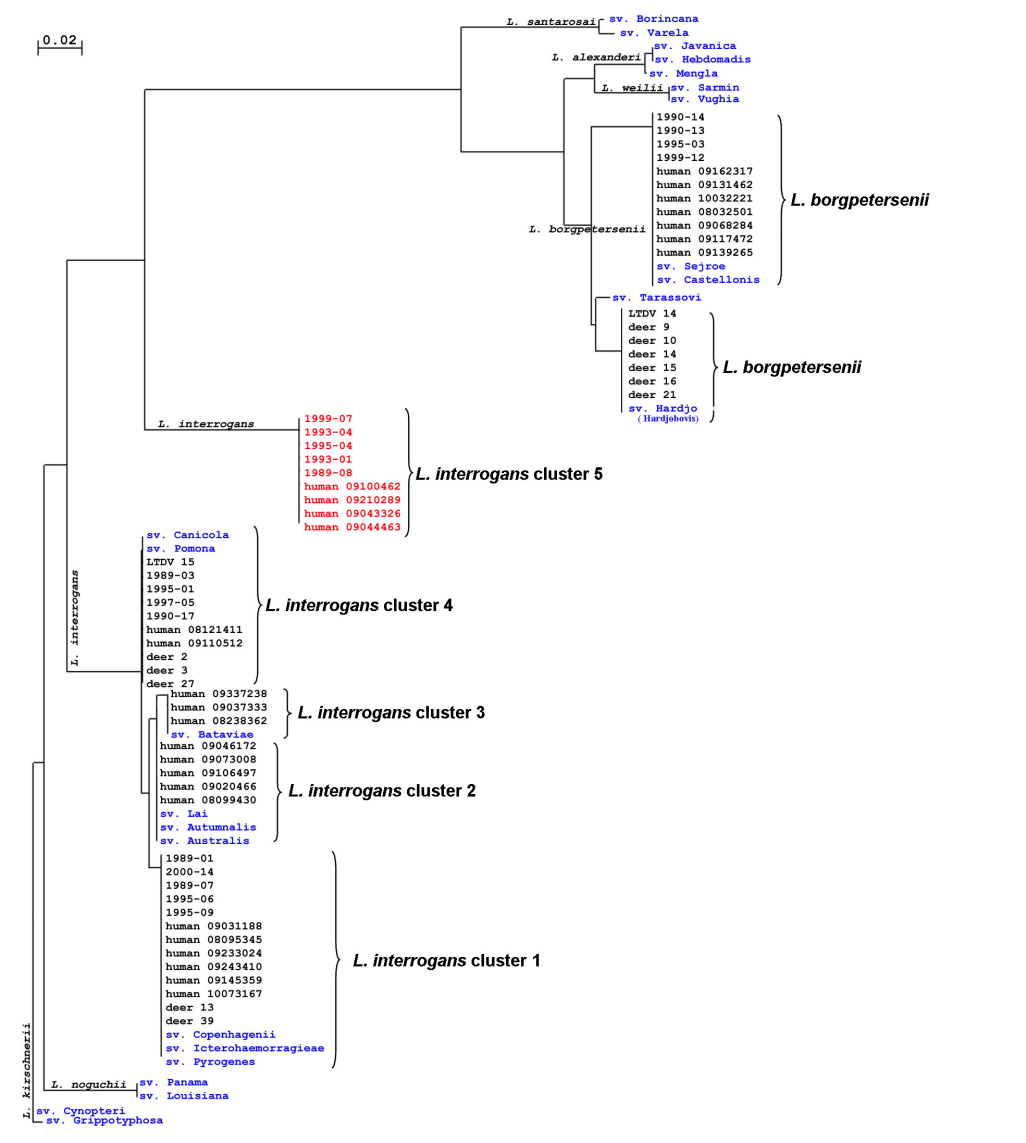
***lfb1*-derived phylogeny of New Caledonian isolates, clinical specimens and reference strains based on a 222 bp sequence polymorphism**. Blue legends indicate reference strains, red legends indicate the putative unknown serovar. GenBank accession numbers are provided as additional file 1 Tables S1 and S2.

These results are summarized in Figure 1 and Table 2 and 4. Among *L. interrogans*-infected clinical samples, five clusters were evidenced: one cluster included the reference strains of the serovars Icterohaemorragiae, Copenhageni and Pyrogenes (later named cluster *L. interrogans *1), one cluster included reference strains of the serovars Lai, Australis and Autumnalis (named cluster *L. interrogans *2), one cluster included the reference strain of the serovar Bataviae (cluster *L. interrogans *3), one cluster included reference strains of the serovars Canicola and Pomona (cluster *L. interrogans *4); lastly, one cluster included no reference sequence of any known serovar (later named *L. interrogans *5).

**Table 4:** Leptospira clusters identified using lfb1 sequence polymorphism.

Clusters	Serovars Reference Strains	Collection isolates	Clinical Samples (number of amplicons)	% of PCR-diagnosed human cases (January 2008-February 2010)
L.int*errogans 1*	Copenhageni/Pyrogenes	5 isolates	Human (60) and deer (2)	68.2%

L.int*errogans 2*	Autumnalis/Australis/Lai	no isolate	Human (6)	6.8%

L.int*errogans 3*	Bataviae	no isolate	Human (3)	3.4%

L.int*errogans 4*	Canicola/Pomona	5 isolates	Human (2) and deer (3)	2.3%

L.int*errogans 5*	Unidentified serovar	5 isolates	Human (10)	11.4%

*L.borgpetersenii 1*	Castellonis/Sejroe	4 isolates	Human (7) and deer (1)	7.9%

*L.borgpetersenii 2*	Hardjo-bovis	1 isolate	Deer (6)	0%

**Figure 2:**
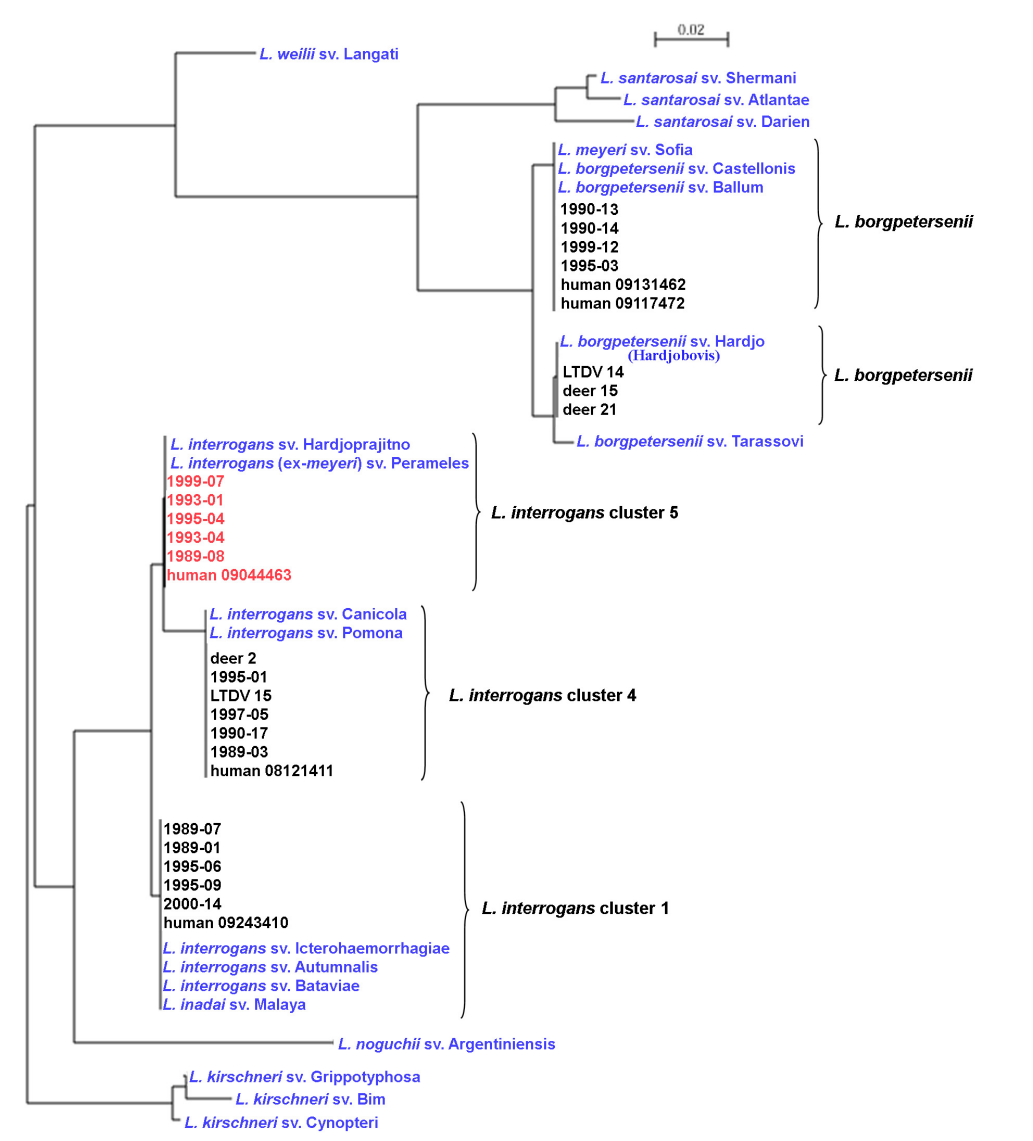
***secY*-derived phylogeny of New Caledonian isolates, clinical specimens and reference strains based on a 174 bp sequence polymorphism**. Blue legends indicate reference strains, red legends indicate the putative unknown serovar. GenBank accession numbers are provided as additional file 1 Tables S1 and S2.

We also evaluated if the direct sequencing of the *secY *diagnostic product [9] could confirm the existence of the different clusters identified using *lfb1 *polymorphism (Figure 2). The 202 bp PCR product could successfully be amplified and sequenced from DNA extracted from all isolates. Using DNA from clinical specimens, samples from both *lfb1*-deduced clusters of *L. borgpetersenii *were successfully amplified and sequenced, but only samples from 3 out of the 5 *lfb1*-deduced clusters of *L. interrogans *could be amplified (clusters *L. interrogans *1, 4 and 5). However, samples from the two remaining clusters (clusters *L. interrogans *2 and 3) were scarce (see Table 4) and had low *Leptospira *concentrations (see Table 2). *secY *products using DNA from these clinical specimens could not be generated, even using combinations of primers used for the MLST study [18] and for diagnosis [9]. However, the phylogeny deduced from a 174 bp alignment of the diagnostic *secY *product confirmed the clusters identified by both the MLST and *lfb1 *typing schemes. Strains from cluster *L. interrogans *5 had sequences 100% identical to *L. interrogans *Hardjo-prajitno (strain Hardjoprajitno) and to *L. meyeri *serovar Perameles strain Bandicoot, a strain recently re-assigned to the species *L. interrogans *[25]. GenBank accession numbers of the sequences generated and used in this study are provided as additional file 1 Tables S1 and S2.

### MLST-deduced phylogeny

**Figure 3:**
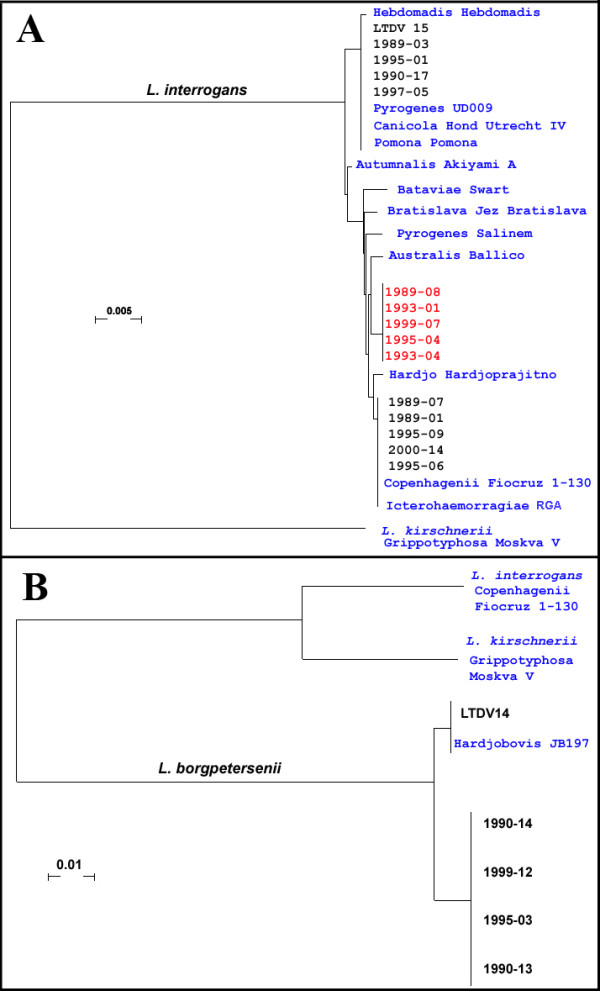
**MLST-deduced phylogeny of New Caledonian isolates and reference strains**. Blue legends indicate reference strains, red legends indicate the putative unknown serovar.. GenBank accession numbers are provided as additional file 1 Tables S1 and S2. **A**: *L. interrogans *phylogeny based on a concatenate 3155 bp sequence. **B**. *L. borgpetersenii *phylogeny based on a *pntA*+*glmU *concatenate 968 bp sequence.

DNA sequences retrieved from databases or sequenced from products successfully amplified were concatenated and allowed drawing a phylogeny of the New Caledonian isolates, together with reference strains (Figure 3). GenBank accession numbers of the sequences generated and used in this study are provided as additional file 1 Tables S1 and S2. Because the DNA sequences of the same reference strains were not available, *secY *sequences were not concatenated with the 7 genes used by Thaipadungpanit et al. [20]. The phylogeny was established independently for *L. interrogans *strains and isolates (7 genes providing a concatenate sequence of 3155 bp) and for *L. borgpetersenii *(2 genes for a total concatenate sequence of 968 bp). Both phylogenies are presented in Figure 3a and 3b respectively. These results evidenced three clusters among the *L. interrogans *New Caledonian isolates and two clusters among *L. borgpetersenii *isolates. Based on sequences of reference isolates available in databases, these clusters could putatively be assigned to a few serogroups. Among *L. interrogans *isolates, one cluster could correspond to serovars Pomona, Canicola, Pyrogenes or Hebdomadis, another one to the serovar Icterohaemorragiae or Copenhageni. Lastly, one *L. interrogans *cluster did not match to any known reference strain. Among *L. borgpetersenii *isolates, one clustered with *L. borgpetersenii *Hardjo-bovis JB197, whereas four other isolates clustered together, but no publicly available sequence allowed putatively identifying this cluster.

### Direct MLST from clinical specimens

To further confirm the existence of the 5 *L. interrogans *clusters identified with *lfb1 *polymorphism on clinical samples, we tried to amplify and sequence *glmU *and *pntA *from these clinical samples, using the MLST primers and PCR conditions. Actually, these 2 genes are correctly amplified from isolates belonging to both *L. interrogans *and *L. borgpetersenii *species and their polymorphism allows discriminating the same clusters within New Caledonian *L. interrogans *isolates as the 7 genes do (data not shown). When using *L. interrogans*-infected clinical specimens, these two genes were successfully amplified from samples infected with ≥ ca. 200 leptospires per ml.

## Discussion

While studying the sequence polymorphism of our diagnostic *lfb1 *qPCR product [15] in clinical specimens and a collection of isolates, we identified 2 *L. borgpetersenii *clusters and 5 *L. interrogans *clusters (Figure 1). Interestingly, one *L. interrogans *cluster (cluster 5) contained only sequences from human clinical specimens and did not include any known sequence of a reference strain, even after extensive searches in public databases.

In order to confirm these presumptive identifications and to try to identify the cluster 5, we then conducted a MLST study using a collection of *Leptospira *isolated in the 1989-2000 period from human leptospirosis cases in New Caledonia, together with two isolates from deer kindly provided by the Veterinary Reference Laboratory. This MLST study similarly evidenced (Figure 3) three clusters of *L. interrogans *(corresponding to isolates grouped in *L. interrogans *clusters 1, 4 and 5). The clustering of isolates was in agreement with the *lfb1*-derived phylogeny. This result suggests that in the New Caledonian context, these *lfb1*-derived *L. interrogans *clusters are monophyletic and probably each correspond to a single serovar. Again, *L. interrogans *cluster 5 did not contain any sequence of a known reference isolate, suggesting that it might correspond to a serovar not yet described, or at least not included in public sequence databases. Though the MLST phylogeny suggests that strains from this latter cluster could be related to the serovar Australis, seroconversions observed in New Caledonian patients infected with this strain merely point to Pyrogenes, a serogroup regarded as serologically related to Australis (data not shown). Whether this cluster corresponds to a serovar not yet described or to a serovar described but which corresponding gene sequences have not been published remains to be studied.

To further identify *L. interrogans *clusters 2 and 3 and to evaluate the feasibility of direct MLST from clinical specimen DNA extracts, we then tried to evaluate the sequence polymorphism of the MLST targets using these clinical samples. Unfortunately, though both *glmU *and *pntA *could successfully be amplified and sequenced from extracts of patients containing ca. 200 leptospires per serum ml or more, none of the patients identified in these 2 clusters had leptospiraemia higher than 50 leptospires per ml. Interestingly, none of the isolate of our collection had *lfb1 *sequences identical to any of these two clusters. Because our isolate collection contains only strains collected until the year 2000, it cannot be known whether strains from these clusters were present in New Caledonia before 2001. They most probably already represented a limited part of the human cases during this earlier period, as suggested by their low incidence over more than 2 years from 2008-february 2010 (see Table 4). It can also be hypothesized that strains from these clusters are of limited virulence to humans, therefore only associated with low leptospiraemia and would therefore seldom be evidenced, either by cultures (before 2001) or PCR (after 2001).

Within *L. borgpetersenii *isolates, only two of the seven genes used in the MLST study of *L. interrogans *could be amplified. Actually, the set of primers used here was described by Thaipadungpanit et al [20] for use in *L. interrogans *isolates and was not supposed to amplify these genes in isolates from other species. Other MLST schemes have been used over a wider range of *Leptospira *species [18,19]. These could have allowed a better typing of New Caledonian *L. borgpetersenii *isolates or clinical specimens. An ongoing program aimed at sequencing the complete genomes of a very large number of pathogenic *Leptospira *isolates (Vinetz J., com. pers.) will allow the selection of the most appropriate targets and to design primers for MLST studies addressing other *Leptospira *species. The phylogeny deduced from the sequence of these 2 genes evidenced two clusters of *L. borgpetersenii*, one including the fully-sequenced *L. borgpetersenii *serovar Hardjo-bovis [26], the other one containing no reference sequence. Again, these clusters were in agreement with the clusters derived from the *lfb1*-based phylogeny. Interestingly, sequences from the cluster containing the Hardjo-bovis reference strain were found only in deer and none of the 88 human clinical samples evidenced this sequence. This suggests that the introduced deer *C. timorensis russa *might be a reservoir for this *Leptospira *strain.

Other gene phylogenies have been studied, demonstrating that these genes might be sequenced to more precisely identify *Leptospira *strains, notably *ligB *[27], *rpoB *[28] and *secY *[8,9,18]. However, though they might prove useful in MLST or other phylogeny studies, most of them can currently only be used when sufficient amounts of DNA of the infecting strain is available, because no high-sensitivity diagnostic PCR was validated using these gene targets. However, a *secY*-based diagnostic PCR was recently described [9] and the sequence polymorphism of the gene segment amplified was validated as a relevant phylogenic tool [8,9]. Therefore, we evaluated if the phylogeny of clinical specimens using this target would confirm the ones obtained with both MLST and the *lfb1 *sequence polymorphism, and notably confirm and provide a more precise identification of *L. interrogans *clusters 2 and 3. The *secY*-derived phylogeny was in agreement with both the MLST and the *lfb1*-derived phylogenies and identified the same clusters (Figure 2). However, *L. interrogans *clusters 2 and 3 that were only evidenced by *lfb1 *polymorphism from clinical specimens could not be confirmed because no *secY *PCR product could be amplified from any of these specimens. Whether this was due to the low leptospiraemia of the corresponding patients (see Table 2) and using a different qPCR platform and different PCR reagents from the ones described by Ahmed et al. [9] or to primer mismatch in the corresponding DNAs remains unknown. Interestingly, *L. interrogans *cluster 5 had a *secY *sequence identical to *L. meyeri *serovar Perameles strain Bandicoot (a strain recently reassigned to the species *L. interrogans *[25]) and *L. interrogans *serovar Hardjo strain Hardjoprajitno. However, this identity was not confirmed by MLST or *lfb1 *sequences.

## Conclusions

Using a combination of MLST and other sequence polymorphisms, we evidenced 7 different *Leptospira *genovars belonging to both *L. interrogans *and *L. borgpetersenii*. They would correspond to at least 7 strains currently circulating in New Caledonia, should two or more strains not be discriminated by this typing scheme. Within these 7 putative strains, one was presumptively identified as *L. borgpetersenii *Hardjo-bovis and could be found only in deer, which might constitute its reservoir. Because deer hunting is a highly frequent practice in New Caledonia both for leisure and subsistence and it can be assumed that hundreds of people are exposed to deer kidneys weekly (frequently bare foot and with no protective gloves), this suggests that this strain is either poorly transmitted, as discussed in light of its genome reduction [26], or of low virulence to humans. We also identified a *L. interrogans *strain (cluster 5) that could not be related to any known reference strain. Though its *secY *sequence suggests that it could be related to known reference strains (*L. interrogans *-formerly *L. meyeri*- sv. Perameles strain Bandicoot and *L. interrogans *sv. Hardjo strain Hardjoprajitno), the more precise MLST sequence polymorphism contradicts this identification. These strains could therefore correspond to a serovar not yet described. We directly amplified two genes of the MLST scheme using extracts from human clinical specimens with leptospiraemia of 200 leptospires per ml or higher. It might therefore be possible to conduct MLST studies directly from clinical specimens if selecting samples with leptospiraemia equal to or higher than 200/ml. Lastly, we demonstrated that the polymorphism of our *lfb1 *diagnostic PCR target is able to provide epidemiologically relevant information, at least in a simple mammal biodiversity context as in New Caledonia. This approach was already proposed using another diagnostic PCR target, namely *secY *[9] that we also evaluated in our study. Using direct sequencing of leptospirosis diagnostic PCR products would partly offset the loss of epidemiological information resulting from the increased use of PCR in the early diagnosis of leptospirosis. This direct typing is currently used in New Caledonia, to better identify the different reservoirs of these *Leptospira *strains. The major mammal species are currently being sampled, in order to better decipher the circulation schemes and reservoirs and adapt prevention measures.

## Authors' contributions

CG conceived the study, coordinated its design, participated in the alignments and phylogeny studies and drafted the manuscript. JP carried out the molecular genetic studies, participated in the sequence alignment and helped drafting the manuscript. Both authors read and approved the final manuscript.

## Supplementary Material

Additional file 1 **Tables S1 and S2**. GenBank Accession Numbers of the nucleotide sequences used in this study. Click here for file
