# Supplementary material for: Rapid Leptospira identification by direct sequencing of the diagnostic PCR products in New Caledonia
Source: BMC Microbiol. 2010 Dec 22;10:325. doi: 10.1186/1471-2180-10-325 (PMC3022709; doi:10.1186/1471-2180-10-325)
Supplement: Additional file 1 — Tables S1 and S2. GenBank Accession Numbers of the nucleotide sequences used in this study. [file 1471-2180-10-325-S1.DOC]

Additional file 1

Title: GenBank accession numbers of the sequences obtained during this study

Description: Table S1 provides the accession numbers of sequences obtained from isolates, Table S2 provides the accession numbers of sequences obtained from clinical specimens.

|  | *lfb1* | *secY* | *pntA* | *glmU* | *tpiA* | *sucA* | *mreA* | *pfkB* | *fadD* |
| --- | --- | --- | --- | --- | --- | --- | --- | --- | --- |
| 1989-01 | HQ328662 | HQ328731 | HQ328697 | HQ328589 | HQ328760 | HQ328745 | HQ328667 | HQ328682 | HQ328574 |
| 1989-03 | HQ328660 | HQ328744 | HQ328698 | HQ328590 | HQ328761 | HQ328746 | HQ328668 | HQ328683 | HQ328575 |
| 1989-07 | HQ328664 | HQ328730 | HQ328699 | HQ328591 | HQ328762 | HQ328747 | HQ328669 | HQ328684 | HQ328576 |
| 1989-08 | HQ328652 | HQ328739 | HQ328700 | HQ328592 | HQ328763 | HQ328748 | HQ328670 | HQ328685 | HQ328577 |
| 1990-17 | HQ328657 | HQ328743 | HQ328703 | HQ328595 | HQ328764 | HQ328749 | HQ328671 | HQ328686 | HQ328578 |
| 1993-01 | HQ328653 | HQ328736 | HQ328704 | HQ328596 | HQ328765 | HQ328750 | HQ328672 | HQ328687 | HQ328579 |
| 1993-04 | HQ328655 | HQ328738 | HQ328705 | HQ328597 | HQ328766 | HQ328751 | HQ328673 | HQ328688 | HQ328580 |
| 1995-01 | HQ328659 | HQ328740 | HQ328706 | HQ328598 | HQ328767 | HQ328752 | HQ328674 | HQ328689 | HQ328581 |
| 1995-04 | HQ328654 | HQ328737 | HQ328708 | HQ328600 | HQ328768 | HQ328753 | HQ328675 | HQ328690 | HQ328582 |
| 1995-06 | HQ328666 | HQ328732 | HQ328709 | HQ328601 | HQ328769 | HQ328754 | HQ328676 | HQ328691 | HQ328583 |
| 1995-09 | HQ328665 | HQ328733 | HQ328710 | HQ328602 | HQ328770 | HQ328755 | HQ328677 | HQ328692 | HQ328584 |
| 1997-05 | HQ328658 | HQ328742 | HQ328711 | HQ328603 | HQ328771 | HQ328756 | HQ328678 | HQ328693 | HQ328585 |
| 1999-07 | HQ328656 | HQ328735 | HQ328712 | HQ328604 | HQ328772 | HQ328757 | HQ328679 | HQ328694 | HQ328586 |
| 2000-14 | HQ328663 | HQ328734 | HQ328714 | HQ328606 | HQ328773 | HQ328758 | HQ328680 | HQ328695 | HQ328587 |
| LTDV15 | HQ328661 | HQ328741 | HQ328716 | HQ328608 | HQ328774 | HQ328759 | HQ328681 | HQ328696 | HQ328588 |
| 1990-13 | HQ328624 | HQ328721 | HQ328701 | HQ328593 |  |  |  |  |  |
| 1990-14 | HQ328625 | HQ328722 | HQ328702 | HQ328594 |  |  |  |  |  |
| 1995-03 | HQ328623 | HQ328724 | HQ328707 | HQ328599 |  |  |  |  |  |
| 1999-12 | HQ328622 | HQ328723 | HQ328713 | HQ328605 |  |  |  |  |  |
| LTDV14 | HQ328626 | HQ328727 | HQ328715 | HQ328607 |  |  |  |  |  |

**Additional Table S1**: GenBank Accession Numbers for New Caledonian *Leptospira* spp. isolates

|  | lfb1 | secY |
| --- | --- | --- |
| 09131462 | HQ328620 | HQ328725 |
| 09243410 | HQ328647 | HQ328717 |
| 09044463 | HQ328627 | HQ328718 |
| 08121411 | HQ328633 | HQ328720 |
| 09117472 | HQ328616 | HQ328726 |
| Deer 2 | HQ328635 | HQ328719 |
| Deer 15 | HQ328612 | HQ328729 |
| Deer 21 | HQ328609 | HQ328728 |
| 09139265 | HQ328615 |  |
| 09068284 | HQ328617 |  |
| 08032501 | HQ328618 |  |
| 10032221 | HQ328619 |  |
| 09162317 | HQ328621 |  |
| 09043326 | HQ328628 |  |
| 09233024 | HQ328629 |  |
| 09210289 | HQ328630 |  |
| 09100462 | HQ328631 |  |
| 09110512 | HQ328632 |  |
| 09046172 | HQ328637 |  |
| 09073008 | HQ328638 |  |
| 09106497 | HQ328639 |  |
| 09020466 | HQ328640 |  |
| 08099430 | HQ328641 |  |
| 08238362 | HQ328642 |  |
| 09037333 | HQ328643 |  |
| 09337238 | HQ328644 |  |
| 09031188 | HQ328645 |  |
| 08095345 | HQ328646 |  |
| 09145359 | HQ328648 |  |
| 10073167 | HQ328649 |  |
| Deer 3 | HQ328634 |  |
| Deer 9 | HQ328610 |  |
| Deer 10 | HQ328613 |  |
| Deer 13 | HQ328651 |  |
| Deer 14 | HQ328611 |  |
| Deer 16 | HQ328614 |  |
| Deer 27 | HQ328636 |  |
| Deer 39 | HQ328650 |  |

**Additional Table S2:** GenBank Accession Numbers for *Leptospira* spp. amplified from New Caledonian clinical specimens
